# Cost reduction strategies in the remediation of petroleum hydrocarbon contaminated soil

**DOI:** 10.12688/openresafrica.13383.1

**Published:** 2022-04-08

**Authors:** Ismail B. Ahmed, Eucharia O. Nwaichi, Ejikeme Ugwoha, John N. Ugbebor, Samuel B. Arokoyu

**Affiliations:** 1 Centre for Occupational Health, Safety and Environment, University of Port Harcourt, Choba, Nigeria; 2 National Oil Spill Detection and Response Agency (NOSDRA), Abuja, Nigeria; 3 Department of Biochemistry, University of Port Harcourt, Choba, Nigeria; 4 Exchange & Linkage Programmes Unit, University of Port Harcourt, Choba, Nigeria; 5 Department of Civil & Environmental Engineering, University of Port Harcourt, Choba, Nigeria; 6 Centre for Research Management and Administration, University of Port Harcourt, Choba, Nigeria; 7 Department of Geography and Environmental Management, University of Port Harcourt, Choba, Nigeria

**Keywords:** Petroleum hydrocarbon, soil remediation, treatment cost, contaminated soil, cost effectiveness

## Abstract

Petroleum hydrocarbon spill on land pollutes soil and reduces its ecosystem. Hydrocarbon transport in the soil is aided by several biological, physical, and chemical processes. However, pore characteristics play a major role in the distribution within the soil matrix. Restoring land use after spills necessitates remediation using cost-effective technologies. Several remediation technologies have been demonstrated at different scales, and research is ongoing to improve their performances towards the reduction of treatment costs.

The process of removing the contaminants in the soil is through one or a combination of containment, separation, and degradation methods under the influence of biological, physical, chemical, and electrically-dominated processes. Generally, performance improvement is achieved through the introduction of products/materials and/or energy. Nevertheless, the technologies can be categorized based on effectiveness period as short, medium, and long term. The treatment cost of short, medium, and long-term technologies are usually in the range of $39 – 331/t (/tonne), $22 – 131/t, and $8 – 131/t, respectively. However, the total cost depends on other factors such as site location, capital cost, and permitting.

This review compiles cost-saving strategies reported for different techniques used in remediating petroleum hydrocarbon polluted soil. We discuss the principles of contaminant removal, performance enhancing methods, and the cost-effectiveness analysis of selected technologies.

## Introduction

Onshore production of crude oil necessitates transportation of petroleum hydrocarbon across vast lands to loading terminals or facilities for further processing. The process of production, transportation, and processing had resulted in several spills either as a result of attacks on facilities or operational reasons, such as equipment failure ^1, 2^. When an oil spill occurs on land, several natural processes are activated, and depending on prevailing environmental conditions, type of oil, spill volume, and soil type, hydrocarbons could percolate into the soil matrix and reach the groundwater ^2^. The migration of oil in the soil is also affected by physical processes such as volatilisation, adsorption, and dissolution in soil pore water. All these factors and time to human intervention determines the extent of the impact on the soil.

Oil spills intensify rapid depletion of nutrients in the soil, thereby rendering it infertile for agricultural purposes ^3, 4^. It also affects the biodiversity of microorganisms in such a way that only tolerant ones are present after the spill ^5, 6^. Oil coats surfaces, so limiting gas and moisture transport across plants. Likewise, the plant is starved of nutrient uptake due to the unfavourable C/N ratio induced by oil. This could stress the plant and possibly lead to its death ^5^. Human exposure to oil spill constituents has been reported to have caused lung diseases, cancer, and skin problems ^5^. Furthermore, run-off from contaminated areas could transport contaminants to nearby water bodies and serve as pathway through which aquatic animals are exposed. Studies have shown the accumulation of polycyclic aromatic hydrocarbons (PAH) in seafood species ^7^. Therefore, an intervention must be made in the form of soil remediation to minimise damage to the environment.

Several methods have been researched and/or implemented in the remediation of petroleum hydrocarbon polluted soil ^8, 9^. While some are still at laboratory scale, there have been records of field applications at large scale. Soil remediation methods are categorised based on the contaminant removal approach and these include containment, separation, and degradation ^10^. Therefore, the choice of remediation technology is determined by other factors, including regulatory clean-up level, permitted time, and cost ^11, 12^.

This review aims to compile cost-saving strategies reported for different techniques used in remediating petroleum hydrocarbon polluted soil. This was based on (1) principles of contaminant removal, (2) performance enhancement method, (3) and cost-effectiveness analysis of selected technologies.

## Transport processes of spilled oil in soil

The cost of soil remediation from the impact of petroleum hydrocarbon is majorly dependent on the volume of the impacted soil and hydrocarbon content. These factors are in turn dependent on the volume of spilt petroleum hydrocarbon and soil type ^13^. The fate of oil in the soil is described by various processes that are categorised as transport, mass transfer, and natural attenuation ^14^. Oil spill in unsaturated soil displaces air from pores and, depending on the connectivity of the pores and gravity head of oil, the migration continues until groundwater is encountered. While oil migration occurs both vertically and horizontally, vertical transport is faster ^15^. When a spill occurs, continuous transport towards the water table is determined by residual retentive capillary forces within soil pores and percolating oil volume head. Depending on the soil profile before the water table, less porous soil, such as clay, may be encountered, which retards downward migration of oil and forms a pool of free-phase oil ^16^. Within porous media that have experienced an oil spill, there may exist sections of residual hydrocarbon held by capillary force, discontinuous free phase within pores held by water films, or pool of free phase due to restricted migration by high capillary pore pressures ^17^.

The transport of oil is accompanied by other mechanisms, such as sorption, dispersion, volatilization, dissolution, and biodegradation ^14^. Sorption of petroleum hydrocarbon to soil particles is influenced by organic matter content, and depending on the age of the spill, bioaccessibility could be hampered ^18– 20^. Soluble fractions of petroleum hydrocarbon could dissolve in pore water and undergo diffusion and dispersion depending on the water flow ^14, 15^. Volatilization of lighter fractions could cause vapour intrusion into nearby buildings or be locked up in soil pores, while biodegradation could occur with or without the presence of oxygen ^16^.

The method and cost of remediation are greatly dependent on the fate of petroleum hydrocarbon described by the transport, mass transfer, and natural attenuation mechanisms. This then requires thorough contaminated soil characterisation to identify the extent and content of petroleum contamination before clean-up or remediation is initiated ^21^.

## Soil remediation technology selection factors

Arriving at the technology in decontaminating petroleum polluted soil involves considering several factors including remediation limit, duration, and cost ^5, 11, 12^.

### Remediation limit

Petroleum facilities traverse several kilometres of land subjected to different uses, and spills at different areas may call for different remediation approaches. Ultimately, the remediation approach could depend on the contaminant level that threatens identified ecological receptors ^22^. All these factors are considered in determining the residual content of petroleum contaminant allowed within an area after a spill. The choice of remediation technology could be determined by the residual level. For remediation of soil contaminated by petroleum hydrocarbon, by Lien *et al.* ^22^ have described that their choice of technology was guided by future land use, which eliminated the risk of receptor exposure through ingestion and dermal contact, which proffered a strategy based on vapour migration only. Duan *et al.* ^23^ suggested that residual level may not be fixed based on total contaminant level but bioavailability, especially when dealing with aged soil contamination. Thavamani *et al.* ^24^ further suggested that other soil physico-chemical parameters could impose toxicity on biota even if residual total petroleum hydrocarbons (TPH) do not, and caution is necessary when fixing remediation limits based on toxicity. Stringent site-specific limits imposed in some sites, such as recreational areas, may warrant the use of technology that achieves a very high performance within a short time or complete removal and replacement of soil.

### Duration

Current or intended land use of the spill area determines how soon the impacted area needs to be remediated, and hence influences the technology to be adopted ^12^. In urban areas with high commercial activities, soil replacement could be the best approach, while in remote rural areas with available land and limited activities, other technology, such as land farming, could be adopted. When time is a primary factor, the cost could play a role when several technologies can achieve remediation objectives within the allowable time.

### Cost

Cost is of the essence in adopting remediation technology. However, this factor plays a role where several technologies are capable of achieving remediation level within the allotted time frame. Several factors determine the cost of remediation and these include treatment mode ( *in-situ* or *ex-situ*), amendment type, energy input, post-remediation waste management, and equipment.

***Treatment mode.*** The cost of excavation and backfilling of soil, as well as haulage between impacted sites and treatment centres, take a chunk of remediation cost if *ex-situ* is an option ^9^. However, this cost could be saved with *in-situ* approaches if effectiveness is guaranteed within the expected period ^25^. *In-situ* microwave heating was demonstrated to treat hydrocarbon polluted soil, and Falciglia *et al.* ^25^ reported an energy cost of €18–27/tonne (t), however Nagkirti *et al.* ^26^ documented $50–330/t for using *ex-situ* thermal desorption.

Depending on the site characteristics, most *in-situ* approaches, such as electrokinetic, bioventing, and soil flushing, could require the injection of enhancers ^8^. However, secondary contamination in groundwater has to be avoided, and if that is not guaranteed, *ex-situ* treatment is usually adopted. In most cases, e *x-situ* has more control over factors that enhance remediation to achieve a higher performance within a short period ^12^.

***Soil amendment.*** Enhancement of remediation performance is usually done through the inclusion of products or energy that aids contaminant separation, fixation, or degradation. However, some remediation methods, such as bioventing, soil vapour extraction or thermal treatment, could proceed without soil amendment. Where an amendment is applied, the nature and cost of the product could increase the general cost of remediation. In the long run, the cost could be offset by increased performance and reduced duration of treatment. In bioremediation, several propriety products are in the market with varying costs ^27^. In a case study reported by Lehr ^27^, a consortium of hydrocarbon utilizing bacteria was employed in remediation of petroleum contaminated soil that was initially earmarked for incineration, and approx. $200,000 was saved after bioremediation. Different from propriety products, the use of organic amendments has also proved successful and cost-effective in soil remediation ^28^.

***Energy input.*** The cost of providing energy to drive the remediation varies with the soil treatment method. The thermal approach of decontamination is considered energy-intensive due to elevated temperatures that need to be achieved for oil desorption and volatilisation ^29^. In aerobic bioremediation where air is needed for effective biodegradation, the energy input could be in form of air supply into the soil matrix within biopile or *in-situ* during bioventing. Other treatment energy input could be in operation of processing or soil handling equipment as well as man-hour. The cost of energy is usually covered under Operation and Maintenance.

***Post-remediation waste management.*** Varying types and quantities of wastes are generated during soil remediation depending on the adopted method. Different wastes recorded in soil remediation include off-gas, wastewater, treatment residue, and other forms of industrial wastes. For example, a high volume of wastewater is generated during soil washing remediation, and it is passed through wastewater processes after the contaminated soil has been cleaned. Also, depending on the soil type handled by the soil washing technique, a significant sludge could be generated, and hence requires further treatment and disposal ^30^.

## Soil remediation technology

Soil remediation takes place on or off-site, through *in-situ* or *ex-situ* technology ^10^. While *in-situ* is carried out on-site, *ex-situ* can be achieved on or off-site. Remediation is regarded on-site, where the impacted soil is decontaminated in position or excavated and treated in an adjoining land, cell or facility within the same site. However, off-site remediation involves excavation of contaminated soil and haul over a distance to a treatment facility ^8^.

**Figure 1.**
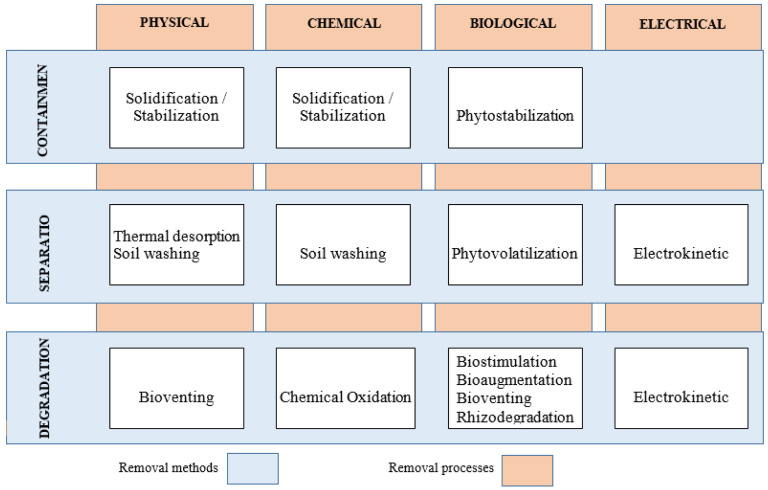
Categorisation of remediation technologies.

As shown in Figure 1, remediation technology can be generally categorised in terms of method (containment, degradation, and separation) and dominant processes of decontamination (physical, chemical, biological, and electrical) ^10, 31^.

### Containment

***Stabilization and solidification.*** Containment involves fixing the contaminant in place, thereby eliminating leaching or migration to receptors. Stabilization and solidification fall in this category, and these are mostly achieved *ex-situ*. However, the products are rarely returned for backfilling. They are diverted for other purposes such as bricks/concrete blocks, road construction, or landfilling ^32^. The operation always involves the physico-chemical process of mixing the contaminated media with other materials, such as binders and other additives, to ensure immobilization of the target contaminants ^32– 34^. In solidification and stabilization practice, the resulting solids must have minimum requirements to qualify them for different final disposal or applications. Crucial requirements include leachability, compressive strength and permeability.

Several binders and stabilizers have been used at different proportions to stabilize petroleum contaminated soil; however, the inclusion of industrial waste materials could be adjudged a low-cost method and waste recycling strategy. Furthermore, using the stabilized solids for different applications, such as civil construction, is an additional waste recycling strategy compared to landfilling. Ahmad *et al.* ^32^ compared the performance of two industrial waste materials, Cement Kiln Dust (CKD) and Limestone Powder (LP), in supplementing cementitious properties of Ordinary Portland Cement (OPC) to stabilize petroleum hydrocarbon contaminated sandy soil. All the proportions of CKD and LP added to 2.5% OPC resulted in permeability and heavy metal leachability below USEPA standard; however, CKD performed poorer than LP in unconfined compressive strength in not meeting USEPA standard of 340 kPa most especially at a hydrocarbon content above 5%. Considering the suitability of the stabilized material for road construction, LP supplements above 20% for up to 10% diesel contaminated sand displayed Unconfined Compressive Strength (UCS) > 1400 kPa, hence could be used as subbase materials for paved and unpaved roads. However, in the case of crude oil contamination, it would take 40% of LP supplement to produce stabilize material with UCS > 700 kPa that could be used as a subbase for only unpaved roads. Ahmad *et al.* ^32^ revealed that the required type and proportions of cementitious materials are determined by the type and concentration of petroleum hydrocarbon in the contaminated soil.

***Phytostabilization.*** Different from the physico-chemical approach of containment, there has been evidence of contaminant stabilization within the rhizosphere of plants employed for phytoremediation ^31^. This could be as a result of plant enzyme influencing the fixation of the contaminant with soil organic matter thereby hampering mobility and bioavailability ^35^. Phytostabilization is common with remediation of metal-polluted soil than petroleum hydrocarbon ^36, 37^. Several phytoremediation studies reported a decrease in TPH within the soil rather than concentrating and fixing it within the soil matrix ^35, 38^.

### Separation

Separation is achieved when petroleum hydrocarbon contaminants are desorbed from soil through a biological, physical, chemical or electrical process. In some situations, further recovery and treatment could be initiated.

***Thermal treatment.*** Thermal desorption technology (TDT) achieves soil decontamination through heat application to facilitate desorption and volatilisation of petroleum hydrocarbon constituents, which are then carried in a stream of sweep gas. The removal efficiency of TDT has been largely dependent on temperature and residence time ^39, 40^. Other factors include heating rate, initial contaminant concentration and sweep gas flow rate ^29^. Due to the binding potential of different soil types, desorption temperature could also be varied for different soils. Leontari *et al.* ^39^ treated petroleum contaminated soil with thermal desorption technology at a varying temperature from 60 to 250°C for 10 and 30 minutes. The study recorded about 94% TPH reduction after residence time of 30 minutes at 250°C, while about 13% TPH reduction was achieved at 100°C at the same time range. However, 10 minutes of holding time recorded less TPH reduction with pronounced difference observed at the higher temperature.

Aside from modification of operational parameters, improved contaminant desorption by the inclusion of additives has been shown to have increased removal efficiency ^41^. Chen *et al.* ^42^ demonstrated the influence of eggshell and plant ash on desorption efficiency during treatment of PAH contaminated soil by thermal desorption. The proportions of the additives varied from 2.5% to 20%, and the mixed soil was subjected to a temperature of 300°C at a residence time of 20 minutes with nitrogen as a carrier gas. Chen *et al.* ^42^ reported the highest desorption efficiencies of 96.6% and 95.9% at 15% plant ash and 10% eggshell inclusion respectively. However, further increase in proportion resulted in reduced efficiency. The performances of the additives were attributed to their improved desorption characteristics, but their poor thermal conductivities contributed to reduced desorption efficiencies when proportions were above optimum.

The type and flow rate of carrier gases can affect the cost and performance of TDT. Carrier gas facilitates the transport of volatilized hydrocarbon from the soil compartment to off-gas treatment facilities. The common carrier gases in use are nitrogen and helium ^29, 41^, but the presence of oxygen can influence the oxidation of hydrocarbon and in turn improve removal performance. Better still, the cost of running TDT may be offset by recycling desorbed volatile organic compounds (VOC) into the combustion chamber of the heating unit.

Performance of TDT has been reported based on removal efficiency without recourse to input energy. Considering offsetting of operational cost, off-gas recovery could be considered an option, provided the recovered gas has an economic value. Several off-gas treatment methods are reviewed by Zhao *et al.* ^29^. Also, the reuse of waste heat could be utilized for several heating purposes ^43^.

***Soil washing.*** Soil washing achieves contaminant separation through solvent interaction with contaminated soil particles. Depending on the solvent used, desorption is initiated and followed by dissolution or floatation for further recovery or treatment of effluent ^44^. Soil washing is done *ex-situ* in a facility, while wash liquid is properly contained and treated before being discharged or recycled. The performance of this technology is influenced by various factors: soil type, initial contaminant concentration, residence time, type of solvent, and mode of treatment ^45, 46^. This method is suitable for sandy or gravel dominated soil, which has less clay/silt and organic matter. However, Li *et al.* ^47^ has reported up to 86% removal performance for illite clay while chlorite dominated clay has a poor removal performance of about 13%. There is a general opinion that bench-scale setup precedes field application to identify the best surfactant for a targeted polluted soil towards cost-saving and achievement of better performance.

Several surfactants have been applied in soil washing technology and they are grouped as cationic, anionic, nonionic, and mixed. However, a mixed surfactant that contains anionic and non-ionic properties have been adjudged better in achieving higher removal performance because of high mixed micelles ^45, 47^. Operational cost recovery is largely covered by hydrocarbon separation or degradation from wash liquid to achieve recyclable solvent ^44, 45^. In a field application of soil washing to remediate 24,620 m ^3^ contaminated soil having an initial TPH of 65,756 mg/kg, Kang *et al.* ^44^ reported reuse of water-based wash liquid after passing through treatment made-up of sedimentation, oil/water separator, air floatation, and activated carbon adsorption. In another field application, reported by Iturbe *et al.* ^46^, water-based surfactant, TW80, was added to water at 0.5% to wash excavated contaminated soil from decommissioned petroleum product distribution and storage site. Rather than ordinary water, TW80 dissolves the petroleum hydrocarbon at every wash. However, the wash water was treated with FeCl _3_ to aid flocculation and sedimentation of surfactant. The treated water was further returned to the plant for further washing. There had been other reports of hydrocarbon degradation from used surfactant, thereby promoting reuse and increase in overall performance per volume of surfactant ^48^.

Rather than dissolution, mobilization and recovery of petroleum hydrocarbon were demonstrated by Wilton *et al.* ^49^ through the use of biopolymer and polystyrene foam pellets (PF) as mobilizers and recovery agents, respectively. 25 mL (1% biopolymer) solvent and 300 mg of PF were mixed with 20 mg contaminated soil and agitated at 200 rpm at 22°C for 30 minutes, after which the floating PF was skimmed off. Wilton *et al.* ^49^ reported higher TPH removal efficiency of 94% at this combination compared to 58% achieved when water and PF were used. Furthermore, the authors suggested cost recovery could be achieved by using the recovered PF as fuel. Also, the biopolymer was derived from plants, while PF was made from recycled material, hence making the method cost-effective.

***Phytoextraction.*** In a plant assisted contaminant separation, phytoextraction describes the translocation of soil contaminants such as petroleum hydrocarbon to the above-ground parts ^50^. The accumulation of the contaminants may occur in the stem and becomes part of plant tissue structure or volatilised through the leaves’ stomata ^51^. The latter is referred to as phytovolatilization and various volatile organic compounds could be removed from soil through this process ^52^. However, integrating contaminant in plant tissue is called phytoaccumulation, and heavier hydrocarbon such as PAH has been reportedly removed through this process ^28, 51, 53, 54^. Nwaichi *et al.* ^28^ studied phytoremediation of crude oil contaminated soil using four different plant species for 90 days. The effect of two soil amendments (cow dung and NPK fertilizer) was also tested. The authors reported improved PAH removal (>80%) from all planted soil. However, amended soil recorded higher removals. Nwaichi *et al.* ^28^ identified plant uptake as one of the removal mechanisms, though soil amendments significantly hampered PAH accumulated by the plants.

Transplanting of plants from one contaminated site to another may be possible provided the accumulation capacity of the plant has not been exhausted. However, plants for phytovolatilization and other processes of phytoremediation could be transplanted across several contaminated media without being stressed as long as toxicity level is not exceeded. Ayuba *et al.* ^53^ reported that the initial concentration of TPH in the soil did not record a significant difference in bioaccumulated content during phytoremediation of crude oil impacted soil. Also, Liao *et al.* ^51^ detected lighter PAH in maize tissue during phytoremediation of crude oil polluted soil, and that the level did not increase with crude oil content in the soil. Possibly transplanting from light impacted soil to heavier impacted soil may not record significant improvement in bioaccumulation, but this requires further study.

Petroleum hydrocarbon constituent accumulation in plants has been established in the literature and is comprehensively reviewed by Hunt *et al.* ^55^. However, evidence of bioaccumulation in animal or human tissue through the ingestion of plants is limited. Therefore, plant recycling by turning it to animal feed may be more sustainable than other conventional treatment, except it has established that petroleum hydrocarbon contaminants could be transmitted up the food chain. Open burning may also not be appropriate because of evidence of PAH release into the soil after bush burning ^56^.

***Electrokinetic.*** Electrokinetic technology involves the introduction of direct current through electrodes into contaminated soil to induce transport of charged particles and/or soil fluid towards electrodes ^57^. The transport of soil fluid facilitates contaminant desorption and migration to the cathode in a process called electroosmosis. However, the removal of hydrophobic constituents of petroleum hydrocarbon is improved by the introduction of surfactant that is capable of solubilizing or aiding desorption of the constituents ^58^. This is one of the strategies of improving electrokinetic performance in remediating petroleum hydrocarbon polluted soil. Other strategies for improved contaminant removal include electrode type and spacing, applied potential difference, polarity switching, and source of energy ^59– 61^. Gidudu and Chirwa ^59^ carried out lab-scale electrokinetic remediation to compare the influence of electrode spacing, voltage supplied, and presence of biosurfactant on oil removal performance from contaminated soil. The authors experimented with 30V and 10V at electrode spacing of 185 and 335 mm arranged in soils contaminated with lubricating oil. In addition, each setup was with or without a biosurfactant. The combined application of 30V, 185 mm electrode spacing and biosurfactant recorded a higher oil recovery of 75%. However, this was achieved at the expense of an increased number of electrodes and energy consumption. Gidudu and Chirwa ^59^ justified the choice of adopting the 30 V and 185 mm configuration with increased performance that offset the increased cost. Invariably this could decrease the period of remediation and time spent on site thereby reduces the amount spent on manpower and machinery ^62^. Detail review of electrode configurations to address pH problems and enhance petroleum hydrocarbon removal from contaminated soil can be found in Saini *et al.* ^61^.

The mode of power supply in electrokinetic has also been reported to have enhanced contaminant removal ^62, 63^. Asadollahfardi and Rezaee ^62^ studied the impact of interrupted and continuous power supply on remediation efficiency of diesel contaminated soil. The researchers used sodium dodecyl sulphate and sodium sulphate as catholyte and anolyte respectively with graphite electrodes. The setup was operated with 1 and 2 V/cm continuously for 7 days, while the same voltages were also operated for 5 days followed by 2 days interruption, and then 2 days reconnection. Asadollahfardi and Rezaee ^62^ reported enhanced removal performance of 57.6 and 63.86% at 1 and 2 V/cm, respectively, for interrupted power supply, while continuous supply recorded 52.48 and 58.62% at 1 and 2 V/cm, respectively. The authors attributed the higher performance of interrupted supply mode to re-equilibration at the solid-liquid interface during the power-off period, and this influenced more dissolution of the hydrocarbon.

### Degradation

***Bioventing.*** Bioventing is an *in-situ* bio-physical activity where air is pumped or injected in subsurface soil to enhance aerobic biodegradation of petroleum hydrocarbon. This technology involves the installation of wells that are terminated within the vadose zone, and the air is injected to enhance the biodegradation of petroleum constituents. The performance of contaminant biodegradation is dependent on other factors, such as available nutrients, distribution of contaminants, moisture content, permeability, and groundwater level ^64– 66^. Preliminary site characterisation conducted by Dominguez *et al.* ^67^ indicated that available nutrient in the soil was in the excess of what would be needed for effective biodegradation, and the only limiting condition was oxygen. In other studies, need for a nutrient supplement may be required to ensure abundance microbes for contaminant degradation. In a pilot-scale bioventing experiment conducted by Mosco and Zytner ^66^, NH _4_Cl was used as a source of nitrogen to stimulate indigenous microbes. Yang *et al.* ^65^ conducted a lab-scale bioventing remediation to study effects of initial concentration, moisture content, CNP ratio, pore volume number, and venting mode on removal performance of diesel from contaminated soil. The authors reported that the driving factors of performance are in the order of; initial concentration, pore volume number, CNP ratio, and moisture content. However, the mode of venting (injection or pumping) recorded the least influence on the TPH removal. Yang *et al.* ^65^ thereafter determined optimum condition for remediating contaminated soil of 40 mg diesel/ g soil as 20% field capacity, 100:20:1, and 4 v _k_/day for moisture, C:N:P, and pore volume number, respectively.

The major factor that drives the budget of bioventing is the cost of energy to drive blowers or air pumps ^67^. Therefore, methods could be designed to minimize this cost. Dominguez *et al.* ^67^ designed and implemented a device that injected air captured from the wind into bioventing wells. The device was able to supply air within the vadose zone to meet up with pre-determined 5% O _2_ utilization per day for effective biodegradation. This was adjudged to have contributed to 90% VOC reduction after the second month of the 15 months operation. The authors reported that the system could have gulped energy of 12,000 kWh/year from the grid if an electric power blower was used. It is a point to note that an adequate preliminary study is necessary to ensure available wind speed within the area will be enough to generate the air pressure required for injection into the wells considering the soil permeability. In addition to air presence, supply rate is also very important to minimise contaminant volatilization, which could increase the cost of off-gas treatment or secondary air pollution. Khan and Zytner ^68^ reduced VOC loss from 2.5% to 0.03% of initial content when the flow rate was reduced from 0.75 mL/min to 0.40 mL/min.

***Bioaugmentation.*** Bioaugmentation is a biological approach of degrading soil petroleum hydrocarbon through the inclusion of exogenous petroleum hydrocarbon degrading microbes (PHDM). This method has proved effective most especially where indigenous microbes are less abundant in achieving the targeted reduction within the allowed time frame ^69^. The PHDM are usually isolated from previous spill areas or oily waste dumps ^70, 71^. Aside from the aforementioned, controlled culture do occur in the laboratory, where colonies of microbes from different sources such as waste treatment plants are treated with petroleum hydrocarbon, and the tolerant ones are isolated for further exposures ^72– 75^.

The PHDM are added to the contaminated media through the inoculum and in some cases, the performance is enhanced by the addition of nutrients in form of fertilizers, or compost. However, there could be initial competition with indigenous microbes ^69, 71^. A means of eliminating such competition was demonstrated by Leal *et al.* ^76^ in the treatment of mature compost with diesel and allowed to stand for 21 days purposely to ensure an abundance of PHDM before mixing with diesel contaminated soil. Also, Nwankwegu and Onwosi ^69^ suggested that immobilized PHDM on biocarriers has improved remediation performance and eliminates competition with indigenous microbes. Zhang *et al.* ^77^ used biochar as a biocarrier in remediating soil contaminated with petroleum hydrocarbon. The authors recorded the highest TPH reduction of 26.85 g/kg in soil treated with immobilized PHDM, while sequential addition of PHDM and biochar resulted in a reduction of 20.74 g/kg. However, PHDM only recorded 17.68 g/kg reduction. The authors suggested that immobilized PHDM were more adapted within a short period and hence improved bioactivity. 

Aside from growing and isolating PHDM before applying to contaminated soil, there have been records of direct treatment with animal manure, and in the course of acclimatization, some microbes die off due to petroleum hydrocarbon toxicity, while others adapted to the new source of carbon ^78^. This method could be considered low cost because the rigorous laboratory culturing and isolation is eliminated. However, the disadvantage of using animal manure include transportation difficulties, inability to store for a long time, and pathogen threat. In addressing the aforementioned, the manure and other organic wastes are stabilized through composting to derive compost rich in nutrients and microbes ^79^. This has been successfully utilized in soil bioremediation and yielded good performance ^79– 82^.

***Biostimulation.*** Biostimulation is a biochemical process of introducing limiting nutrients required by PHDM into the petroleum-contaminated soil for enhanced biodegradation. Several sources of nutrients have been developed and tested at different scales, and reportedly achieved higher TPH degradation compared to natural attenuation ^83^. Spill areas that consist of indigenous microbes would require microbial stimulants to sustain growth towards enhanced hydrocarbon removal. Soil characterisation would reveal limiting nutrient that needs to be supplemented to avoid excessive products that may inhibit the growth of degrading microbes. The CNP ratio commonly adapted for effective growth of PHDM are 100/5/1 and 100/10/1 ^75, 77, 84^. Koolivand *et al.* ^85^ investigated how varying CNP ratios affected TPH removal in the treatment of oily sludge. After 12 weeks of composting setup with CNP ratios of 100/5/1 and 100/10/1, the authors did not record a significant difference in the TPH. However, Koolivand *et al.* ^85^ proposed adoption of 100/5/1 for an economical reason as less nutrient is required. There are several nutrient propriety formulated products for bioremediation of petroleum impacted soils ^27^. These products contain mainly nitrogen and phosphorus with or without other simple organic compounds in different combinations. Considering the less expensive approach of bioremediation, animal manure, sewage sludge and organic waste materials have also been adjudged as effective biostimulants ^78, 86– 88^. However, Cunningham *et al.* ^86^ cautioned against the use due to them being potential sources of antibiotics resistant genes (ARGs) that could enter food chain. Several studies have shown that organic waste composting goes a long way to reduce ARGs, and compost could be considered safer as biostimulants in bioremediation ^79, 89^

***Rhizodegradation.*** Tolerant plant roots have been implicated in secreting exudates that stimulate microbes within the rhizosphere to degrade petroleum hydrocarbons ^38, 90^. This is regarded as one of the processes of phytoremediation, and it is called rhizodegradation. The exudates released into the soil contain organic nitrogen, enzymes, simple organic acids, and organic compounds that have a similar structure with some petroleum hydrocarbon constituents ^38, 91^. With this, several mechanisms are suspected to be responsible for TPH degradation based on exudates composition. Among these include the release of enzymes, such as Laccases and Peroxidases, that enhance the bioavailability of petroleum hydrocarbon constituents. Similarly, simple organic acid desorbs strongly adsorbed TPH constituents from soil particles and hence improve microbial degradation. Co-metabolism could also be induced by exudates constituents that mimic the structure of petroleum hydrocarbon constituents such as PAH. Several plants have been demonstrated to have the potential of TPH removal from petroleum contaminated soil through these processes, however, using indigenous plant species is preferable to avoid the introduction of invasive plants ^28^. In addition, indigenous plants have existing adaptation to soil microbiomes, which is an important factor in phytoremediation ^38^. Lastly, the cost of transportation and preservation could discourage the introduction of foreign plants.

TPH removal through rhizodegradation by exudate-stimulated microbial-plant root nexus has been adjudged a time-consuming process. In addition, the radius of influence is few centimetres away from the root, and also the performance decreases with distance from the root ^92^. Several efforts have been demonstrated to have improved the performance of this process. These include the introduction of biostimulants ^28, 38, 93, 94^, biosurfactant ^38^, or plant-growth enhancing microbes ^38, 92, 95^. Seo and Cho ^94^ investigated the influence of soil amendments (compost and NPK formulation), and plants (maize and tall fescue) on the reduction of TPH from artificially contaminated potting soil. The authors reported a lag phase of 12 days in unplanted pots when insignificant TPH change was recorded. However, planted pots recorded a rapid drop in TPH and higher removal performance after the experiment. Moreover, compost amended pots showed improved performance than a chemical nutrient, and this was attributed to exogenous microbes and nutrients introduced into the rhizosphere. Nwaichi *et al.* ^28^ also reported enhanced phytoremediation in the presence of poultry dung and NPK fertilizer as amendments. The authors attributed the higher performance of animal manure to increase organic matter content that invariably led to improved bioactivity.

***Modified electrokinetic remediation.*** The principle of electrokinetic remediation is based on the mobility of soil fluids. However, the degradation of petroleum hydrocarbon contaminants is facilitated by the introduction of fluids that contain microbial limiting nutrients, hydrocarbon degraders, biosurfactants, or oxidants ^96– 100^. Fan *et al.* ^101^ mixed PHDM suspension with artificially contaminated soil and subjected it to an electrokinetic setup (B-EK). Other setups the authors studied included electrokinetic in contaminated soil untreated with microbial suspension (EK), soil treated with microbial suspension but no electrokinetic arrangement (B), and control under natural attenuation (NA). Fan *et al.* ^101^ attributed the highest TPH removal by B-EK to improve mixing and distribution of PHDM and contaminants during electromigration as polarity changes. However, the TPH removal performance was spatially different within the soil matrix of EK and B-EK setups. The highest performance was recorded close to the electrodes. At the end of 98 days, Fan *et al.* ^101^ recorded TPH removal performance of 8.8, 36.6, 47.7, and 72.3% from NA, B, EK, and B-EK respectively. It is a point to note that the increased cost associated with the provision of PHDM in the experiment could be offset by the increased performance, which could be a justified reason.

Aside from soil amendments, electrode type has a major influence on the cost and performance of modified electrokinetic remediation. In the Fenton modified electrokinetic system demonstrated by Adhami *et al.* ^99^ using copper or iron electrode, higher TPH removal from oil base drilling mud was recorded for the Fenton modified system compared to conventional electrokinetics. The authors attributed the performance of the Fenton modified system to the initial oxidation of heavy hydrocarbon by radicals produced in anolyte spiked with hydrogen peroxide, which further increased the biodegradability of the intermediates. The Fenton modified electrokinetic recorded TPH removal of about 120% higher than what conventional electrokinetic recorded. However, while Fenton modified system with copper electrode caused an increase in energy consumption by 9.27% compared to conventional, the modified system with iron electrode recorded less energy consumption by 20% relative to the conventional one. The iron electrode, nevertheless, corroded due to low pH and was deposited within the electrode compartment. This is of concern, especially when considering *in-situ* applications in the field.

## Cost effectiveness analysis

The effectiveness of technology in remediating petroleum contaminated soil is not defined in isolation without considering parameters that define the acceptability of the work. Local Regulatory Standards play a major role in this aspect by specifying allowable limits and periods at which a contaminated site is to be remediated. While several technologies are effective in delivering the work within the required time, a remediation engineer still considers cost in selecting the appropriate method. Table 1 shows varying performances of different technologies with different effective periods, and it is obvious that high efficiencies are achieved at varying periods. Several studies have compared different technologies that have wide effective periods ^102^. In Table 1, all remediation technologies that involve enhanced natural processes achieved good performance after several days or months, while other processes such as thermal, soil washing, or chemical oxidation achieved high performance within a very short time. Meuser ^12^ categorised remediation periods based on time to decontaminate 3 ha of land, and these are; “weeks to few months”, “more than 4 months to 2 years”, “3–10 years” and “decades”. Rather, this review considers the period to remediate a unit volume of soil, and therefore categorised as short term (< 1 day), medium term (several days but less than one month), and long term (> 1 month). Furthermore, all cost analyses are uniformly expressed in USD/t assuming soil bulk density of 1500 kg/m ^3^. Where necessary, currency conversion at the rate of €1.19/USD was adopted.

**Table 1.** Remediation enhancement strategies and their performances.

Category	Process	Soil qty	Performance enhancement strategy	Efficiency (%)	Period	Reference
**Degradation**	Chemical oxidation	10 g	Persulphate activated by ultrasonic and zero valent iron	56.28	3 d	Li *et al.* ^103^
**Degradation**	Chemical oxidation and bioaugmentation	2.3 kg	Hydrogen peroxide + Cultured hydrocarbon-degrading bacteria (Acinetobacter sp.) + NPK fertilizer	86	30 d	Bajagain *et al.* ^75^
**Degradation**	Solvent extraction + chemical oxidation	5 g	Ethyl lactate + Hydrogen peroxide	96.8	4 h	Jalilian Ahmadkalaei *et al.* ^104^
**Degradation**	Bioaugmentation	500 g	Cultured hydrocarbon-degrading bacteria immobilised on biochar	56.2	60 d	Zhang *et al.* ^77^
**Degradation**	Phytoremediation	13,000 m ^2^	Plant growth-promoting rhizobacteria			Murray *et al.* ^95^
**Degradation**	Chemical oxidation	20 g	Persulphate+Soil minerals	94.14	48 h	Satapanajaru *et al.* ^105^
**Degradation**	Bioaugmentation and biostimulation	1 kg	Hydrocarbon degrading consortium + NH _4_NO _3_ and K _2_HPO _4_	5	112 d	Wu *et al.* ^106^
**Degradation**	Biostimulation	500 g	Intermittent spray NH _4_NO _3_ and K _2_HPO _4_	53.5	90 d	Wu *et al.* ^84^
**Degradation**	Bioaugmentation and biostimulation	1 kg	Hydrocarbon degrading consortium + NH _4_NO _3_ and K _2_HPO _4_	93.14	42 d	Varjani *et al.* ^107^
**Degradation**	Chemical oxidation	10 g	Ultraviolent + ultrasonic + Fenton process	99	3 h	Gharaee *et al.* ^108^
**Degradation**	Phytoremediation and Electrokinetic	4 kg	Reverse polarity	84	20 d	Rocha *et al.* ^109^
**Degradation**	Bioaugmentation	250 tons	Hydrocarbon degrading bacteria consortium	95.9	63 d	Poi *et al.* ^74^
**Degradation**	Biostimulation	2 kg	Poultry manure + plam bunch ash + granite dust	96.7	70 d	Chikere *et al.* ^78^
**Degradation**	Biostimulation	50 g	Biochar + N (Urea) + rhamnolipid	80.9	50 d	Wei *et al.* ^110^
**Degradation**	Biostimulation	250 g	Poultry manure + Avocado peer seed cake	89.6	70 d	Olatunji *et al.* ^111^
**Degradation**	Biostimulation	150 g	Poultry manure	73.4	8 wks	Aghalibe *et al.* ^112^
**Degradation**	Bioaugmentation and biostimulation		Immature compost	79.5	16 wks	Farzadkia *et al.* ^81^
**Degradation**	Bioaugmentation and biostimulation	60 g	Diesel modified compost + NPK	99.4	20 d	Leal *et al.* ^76^
**Degradation**	Bioaugmentation and biostimulation	3 kg	Autoclaved compost + Petroleum degrading bacteria	84.3	12 wks	Abtahi *et al.* ^71^
**Separation**	Thermal desorption (fast pyrolysis)	10 g	Nitrogen carrier gas and temperature of 500°C	100	5 mins	Li *et al.* ^113^
**Separation**	Soil washing	20 g	Biopolymer + Polystyrene Foam Beads	94	30 mins	Wilton *et al.* ^49^
**Separation**	Microwave	30 g	Spent graphite as a susceptor	91.18	60 mins	Sivagami *et al.* ^114^
**Degradation**	Phytoremediation		Harvested and replanted	56	100 d	Mita *et al.* ^115^
**Degradation**	Vermiremediation	1 kg	Earthworm	84.99	90 d	Ekperusi and Aigbodion ^116^
**Degradation**	Bioelectrochemical	340 g	Glucose			Li *et al.* ^117^
**Degradation**	Phytoremediation	2 kg	Compost	ca. 75.5	79	Seo and Cho ^94^
**Degradation**	Electrokinetic		Surfactant Sodium dodecyl sulphate as catholyte and sodium sulphate as anolyte	63.86	9	Asadollahfardi and Rezaee ^62^

### Short term

Some remediation technologies such as thermal desorption and soil washing are capable of delivering high removal performances within minutes or hours ^44, 49, 104, 108, 113^. These have been applied at various scales and proved effective within the context in which they were tested. In the *ex-situ* approach, the processes involved include excavation, transportation, treatment, backfilling/landfilling, and waste management. However, the *in-situ* application, which involves only treatment and waste management, is considered less costly, but is disadvantaged due to non-uniform performance due to heterogeneity of soil ^8^. Therefore, the common activities for all the technologies are treatment and waste management. The thermal approach generates gaseous waste, which is handled by an off-gas treatment setup. However, soil washing generates liquid waste that is handled by wastewater treatment processes. For example, chemical oxidation generates much less treatment waste compared to others; therefore, its cost of waste management could be less. *Ex-situ* chemical oxidation is a fast degradation process depending on the oxidant employed, though several activating agents had been used to enhance the performance ^103, 105, 118^. Moreover, soil treatment costs may encompass associated waste decontamination, and separate quotation may not be presented for off-gas or wastewater treatment ^30^.

In thermal treatment, several factors could determine cost of decontamination. Hyman and Dupont ^30^ factored in moisture effect on cost and quoted a range of $52 – 69/t depending on moisture content between 10 and 30% for about 4,500 tonnes of contaminated soil. Lehr ^27^ quoted a range of $55 – 138/t for treatment cost that excludes excavation and permitting. Meuser ^12^ reported an estimate between $23 and $152/t for thermal remediation.

In soil washing, cost includes wastewater treatment but not sludge disposal, and it ranges between $88 and $276/t depending on whether the soil is co-contaminated with other pollutants such as heavy metals ^30^. Lehr ^27^ reported a range of $110 – 221/t and the cost is dependent on proportions of silt, clay, and organic content. Meuser ^12^ quoted a range of $53 – 76/t.

The treatment costs for thermal desorption and soil washing are relatively within the same range. However, sludge disposal is not captured for soil washing cost analysis, and depending on the type of soil being treated, sludge volume could be significant ^44^. This may invariably cause an increase in the cost. For thermal desorption, it was suspected that higher oil content in soil could result in reduced cost of treatment due to reduced fuel consumption, while moisture content could lead to an increase ^30^. For coarse sandy or gravel contaminated media that generates less sludge, soil washing could be preferred, especially when an existing hazardous wastewater treatment plant is nearby to reduce the cost of setting up an on-site plant.

### Medium term

Several technologies such as modified electrokinetic remediation, bioremediation, *in-situ* chemical oxidation (ISCO), as shown in Table 1, achieve high performance in less than a month ^76, 98, 105^. Table 1 shows study periods at which different technological performances were reported.

Bioremediation is perceived to take a long time in decontaminating polluted soil, but recent improvements in biostimulation and bioaugmentation, as well as active aeration, have been changing the narrative ^76^. Likewise, the performance of electrokinetic remediation has recently been improved by integration with other methods such as phytoremediation ^109^, chemical oxidation ^100^, and bioremediation ^98^. On the other hand, ISCO may take a longer time to achieve high removal performance because of soil permeability and the uneven distribution of contaminants in comparison with the *ex-situ* approach that is faster due to improved soil-oxidant homogenisation ^119^.

Lehr ^27^ projected that the cost of electrokinetic remediation could be within $44 - 131/t. For composting, Lehr ^27^ reported a range of $22 - 88/t. These costs depend on initial concentration, clean-up level, soil composition, type of amendments, utilities, sampling and analysis among others. ISCO depends on oxidants in use, and Aurora and Juana ^119^ estimated between $9 – 80/t. Also, Lehr ^27^ quoted a range of $22 – 66/t for ISCO.

### Long term

Some remediation technologies are effective after several months or years. Notably among these are natural processes dependent approaches such as bioventing, biopiling, landfarming, and phytoremediation. However, several efforts have been demonstrated to enhance their performances ^77, 84, 120^.

Bioventing is usually operated for more than one month depending on the area of impact and scale ^25^. Dominguez *et al.* ^67^ reported the operation of wind-driven bioventing for 13 months. For bioremediation approaches such as biopiling, landfarming, and composting that adopt biostimulating and/or bioagmenting agents to enhance the performance of contaminant removal, their operations can take few months in treating a batch of soil to achieve clean-up targets. Phytoremediation application takes years to reach the clean-up level depending on the initial concentration. However, its application in petroleum hydrocarbon polluted soil is less expensive compared with other contaminants such as metals or radioactive elements ^27^.

The cost of bioventing is mostly dependent on mode of introducing air into the soil, actively or passively ^121^. Dominguez *et al.* ^67^ saved equivalent energy cost of 20 kWh/year when active wind driven equipment was used to supply air. Generally, Lehr ^27^ estimated a cost range of $7 – $20/t for bioventing with soil volume below 11,000 m ^3^. Meuser ^12^ reported a common range of $14 – 40/t.

In a bioremediation pilot study conducted by Ighilahriz *et al.* ^122^, treatment cost was estimated at $13/t of oily sludge. For the use of some propriety products, Lehr ^27^ quoted treatment cost of $18 – 131/t for bioaugmentation products, while biostimulators are between $13 – 79/t. Meuser ^12^ recorded a range of $21 – 119/t for landfarming and biopiling.

Gerhardt *et al.* ^123^ reported a range of $28 – 111/t for general phytoremediation remediation. Furthermore, the authors estimated reduced range $13 – 26/t when plant growth promoting rhizobacteria enhanced phytoremediation is adopted. Lehr ^27^ reported a range of $11 – $39/t.

## Conclusion

Different land use warrants different approaches of remediation when spills occur. The need to save costs while achieving remediation goals has necessitated different performance enhancement strategies for remediation technologies. Importantly, enhancement is in the form of speeding contaminant separation, degradation or containment through physically, electrically, chemically, or biologically dominated processes. In most cases, product or energy is introduced into the contaminated soil matrix to enhance pollutant’s desorption and dissolution or volatilization towards facilitation of free phase separation, oxidation or degradation. Despite the improvement, the period of effectiveness varies and could be categorised as short, medium, and long terms. For example, thermal desorption, soil washing, and chemical oxidation are capable of achieving high removal performance within a few minutes or hours and are categorised as short term technologies. However, contaminated soil characteristics, clean-up level, and site location may determine additional cost of treatment and the final choice of technology.

Generally, short term technologies have treatment costs in the range of $39 – 331/t, excluding excavation, transportation, permitting, samples analysis, equipment rentals, and other special activities. On the other hand, the medium-term technologies achieve high removal performance after several days within a month. These include modified electrokinetic remediation, in-vessel composting, and *in-situ* chemical oxidation. These technologies have a treatment cost range between $22 – 131/t, excluding other capital costs. Lastly, long term technologies, such as phytoremediation, biopiling, landfarming, and bioventing, could incur treatment costs in the range of $8 – 131/t of a contaminated site. In comparing technologies in terms of cost-effectiveness, it is suggested that comparison be made within respective effective period.

## Data availability

No data are associated with this article.
