# Supplementary material for: Cost reduction strategies in the remediation of petroleum hydrocarbon contaminated soil
Source: Open Res Afr. 2022 Apr 8;5:21. [Version 1] doi: 10.12688/openresafrica.13383.1 (PMC9718438; doi:10.12688/openresafrica.13383.1)
Supplement: Peer review history

# Peer review: Reviewer response for version 1

**DOI:** 10.21956/openresafrica.14531.r29399

**Published:** 2022-11-14

**Authors:** Chukwuma Ogbonnaya

**Affiliations:** 1 Department of Mechanical, Aerospace and Civil Engineering, The University of Manchester, Manchester, UK

The article is well presented and Table 1 is very valuable in substantiating the title of the article. However, I recommend the following for the authors to consider:

1. At the Introduction Section, the authors should highlight the contribution of the study. Why is this work worth publishing? Justify the new ideas in the article beyond compiling cost saving strategy for remediation.
2. It appears that there is a need to state the outline/overall structure of the article at the end of the introduction section. There were some sections that were not completely on cost and readers may need to get an overview.
3. Can you be more explicit on what you mean by "fate of petroleum hydrocarbons" on page 2. It was used twice.
4. Can you create a section before the conclusion to present the research gaps that other researchers can consider?  In other words, what are the effective cost reduction strategies that are yet to be fully explored? Or other aspects beyond cost that should be explored. Comment on the roadmap for future research regarding the topic of the review.

Overall, the paper is an interesting read.

Is the review written in accessible language?

Yes

Are all factual statements correct and adequately supported by citations?

Yes

Are the conclusions drawn appropriate in the context of the current research literature?

Yes

Is the topic of the review discussed comprehensively in the context of the current literature?

Yes

Reviewer Expertise:

Energy, Manufacturing, Sustainability, Polymers, Engineering

I confirm that I have read this submission and believe that I have an appropriate level of expertise to confirm that it is of an acceptable scientific standard.

# Peer review: Reviewer response for version 1

**DOI:** 10.21956/openresafrica.14531.r29196

**Published:** 2022-05-26

**Authors:** Mojisola Christiana Owoseni

**Affiliations:** 1 Department of Microbiology, Federal University of Laﬁa, Lafia, Nigeria

The review of cost reduction strategies in the remediation of petroleum hydrocarbon contaminated soil expounds on the various cost reduction strategies available for effective remediation of soils. The review was well written providing adequate information.

 Statements were supported with citations and the reader can connect each remediation approach to the cost of each technique. The conclusion compares the cost within each group of strategies.

 The authors should address the following areas:

1. The authors should provide possible comparisons among the various cost-reduction strategies to enable a robust conclusion to be drawn in relevance to the topic.
2. The authors expatiated on the effect of the soil type and other factors on the treatment strategies. Considering the climatic and economic constraints  influencing cost effectiveness of each treatment strategy in each environment, can the authors discuss regions or areas where each cost-reduction strategy can be applicable and the reasons for their specifications?

Is the review written in accessible language?

Yes

Are all factual statements correct and adequately supported by citations?

Yes

Are the conclusions drawn appropriate in the context of the current research literature?

Yes

Is the topic of the review discussed comprehensively in the context of the current literature?

Yes

Reviewer Expertise:

Environmental microbiology and public health, remediation, water quality.

I confirm that I have read this submission and believe that I have an appropriate level of expertise to confirm that it is of an acceptable scientific standard.
